# Remote physical activity intervention to promote physical activity and health in adolescent girls (the HERizon project): a multi-arm, pilot randomised trial

**DOI:** 10.1186/s12889-024-19664-7

**Published:** 2024-08-03

**Authors:** Emma S. Cowley, Paula M. Watson, Craig Paterson, Anton J.M. Wagenmakers, Andrew Thompson, Sarahjane Belton, Dick Thijssen, Lawrence Foweather

**Affiliations:** 1 Research Institute of Sport and Exercise Science, Liverpool John Moores University, Liverpool, UK; 2 Population Health Science, Bristol Medical School, University of Bristol, Bristol, UK; 3 Wolfson Centre for Personalized Medicine, Institute of Systems, Molecular and Integrative Biology, University of Liverpool, Liverpool, UK; 4 School of Health and Human Performance, Dublin City University, Dublin, Ireland; 5 Radboud Institute of Health Sciences, Department of Physiology, Radboud University Medical Centre, Nijmegen, The Netherlands

**Keywords:** Physical activity, Adolescents, Girls, Intervention study, Behaviour change, Remote

## Abstract

**Background:**

Engaging in physical activity (PA) during adolescence is beneficial for health and positive development. However, most adolescent girls have low PA levels, and there is a need for interventions outside of school hours. This pilot randomised controlled trial aimed to explore the preliminary effectiveness of three different remote PA interventions in increasing adolescent girls’ moderate-to- vigorous PA (MVPA), fitness and psychosocial outcomes.

**Methods:**

Girls living in the UK or Ireland, aged between 13 and 16 years old, who wished to increase their activity levels, were eligible for the study. Using a random number generator, participants (*n* = 153; 14.8y ± 1.4) were randomised into one of three 12-week intervention groups (i) PA programme, (ii) Behaviour change support, or (iii) Combined PA programme and Behaviour change support, or (iv) a Comparison group. Outcome measures included accelerometer and self-reported PA, physical fitness (cardiorespiratory fitness; 20 m shuttle run, muscular endurance; push up, muscular strength; long jump), and psychosocial assessments (perceived competence; body appreciation; self-esteem; behavioural regulation). Linear mixed models were used to analyse differences between each intervention arm and the comparison group immediately postintervention (12 weeks) and at follow up (3-months post-intervention), while adjusting for potential confounders.

**Results:**

Participation in the PA programme group was associated with higher perceived competence (0.6, 95% CI 0.1 to 1.2), identified regulation (0.7, 95% CI 0.2 to 1.1) and intrinsic motivation (0.9, 95% CI 0.2 to 1.6) at post-intervention. Participation in the Behaviour change group was associated with higher perceived competence at post-intervention (0.6, 95% CI 0.1 to 1.2), and higher push-up scores at the 3-month follow-up (4.0, 95% CI 0.0 to 7.0). Participation in the Combined group was also associated with higher perceived competence at post-intervention (0.8, 95% CI 0.2 to 1.4), and higher push-up scores at the 3-month follow-up (5.0, 95% CI 1.0 to 8.0). No other significant differences were found between the intervention arms and the comparison group.

**Conclusion:**

Results suggest perceived competence increased across all intervention arms, while the PA programme group enhanced autonomous motivation in the short term. Intervention arms with behaviour change support appear most promising in improving muscular endurance. However, a larger scale trial is needed for a better understanding of between-group differences and the impact of intervention arms on MVPA and fitness, given the small sample size and short-term follow-up.

**Supplementary Information:**

The online version contains supplementary material available at 10.1186/s12889-024-19664-7.

## Introduction

Physical activity (PA) levels in adolescent girls are low. Despite the overwhelming evidence supporting the physical [1], psychological [2], and social benefits of PA [3], approximately 80% of teenage girls in the United Kingdom (UK) and Ireland do not meet the World Health Organisation minimum PA participation guidelines of an average of 60 min daily moderate-to-vigorous physical activity (MVPA) [4]. Further, PA is found to decrease at a more rapid rate as girls progress through adolescence. For example, there is an annual decrease of 3% in MVPA for girls from 12 years of age, which accelerates to a 13% decrease by 15 years [5]. Individuals who engage in high levels of PA during adolescence are found to have better health behaviours than their inactive counterparts in adulthood (6–7). To combat this persisting health concern, more focus has been placed on female-specific PA interventions in recent years [8–12], alongside the introduction of policies and laws such as Title IX (America) and The Equality Act 2010 (UK).

Given adolescents spend a high proportion of their waking time in school, it is not surprising that the school environment has been the primary setting for interventions attempting to modify adolescent PA behaviour (13–14). Yet, a 2019 meta-analysis found school-based interventions have little-to-no effect on youth MVPA [15]. These null results may be due to difficulty in standardised intervention implementation across schools, classrooms, and teachers [16], as well as the variance in resource availability, space within the timetable to dedicate to research, and the priorities of the school/ individual teachers. The decline in PA among youth predominantly happens outside of school [17]. This emphasises the importance of considering settings beyond the school, such as the home or community [18], when designing interventions to increase adolescents’ MVPA.

Interventions informed by behaviour change theory are thought to be more effective than those without [19]. Self-determination theory (SDT) [20] is commonly used in PA interventions to promote intrinsic motivation. To obtain optimal wellbeing and social development, SDT posits three psychological needs must be met; autonomy (i.e., perceived control), competence (i.e., perceived ability to carry out a task), and relatedness (i.e., feeling connected to others) [20]. These needs are also commonly cited factors influencing adolescent girls’ engagement in PA [21]. For example, adolescents are more committed to a PA intervention when they are given autonomy and a sense of ownership [22], girls are more likely to drop out of sport if they feel insecure about their abilities [23], and girls who feel supported by peers, family and friends are more likely to be physically active [21]. SDT has been used in many school-based interventions with varying degrees of success in increasing girls’ PA (9–10, 24). To the best of the authors knowledge, there are no remote, home-based PA interventions that utilise SDT specifically for adolescent girls. However, research conducted with other populations has shown promising results. For example, participants in a 12-month remote SDT intervention had significantly improved MVPA in comparison to traditional health behaviour group [25]. Further, adults with spinal cord injury in a SDT group had significantly greater autonomous motivation and leisure-time PA in comparison to the control group [26]. With this in mind, it was hypothesised that a remote, home-based intervention founded in SDT and that aims to meet girls’ basic psychological needs could effectively increase PA.

The HERizon Project was a remote PA intervention founded in SDT [20] that aimed to increase adolescent girls’ MVPA by employing behaviour change strategies such as guided choice, goal setting, self-monitoring, and planning. The intervention components included weekly behaviour change support calls, weekly online live group PA sessions and an online group community. HERizon followed the Medical Research Council (MRC) iterative phased approach to the development and evaluation of complex interventions (27–28), with initial qualitative work informing intervention development [29], a small-scale formative evaluation assessing the interventions’ feasibility [30], and a comprehensive process evaluation exploring the implementation and acceptability of the intervention [31]. Results of the formative evaluation suggest HERizon has a positive effect on girls’ intrinsic motivation towards PA, self-esteem, and body appreciation [30], but it was unclear which intervention components contributed to these promising results. Such information would be valuable for informing future implementation and scale-up to a larger, definitive trial. Consequently, a subsequent exploratory multi-arm pilot randomised controlled trial was designed to examine specific effect of the intervention components, specifically (i) a physical activity programme, (ii) behaviour change support, or (iii) the combination of physical activity programme and behaviour change support. This study aimed to examine the preliminary effectiveness of specific HERizon intervention components on increasing adolescent girls’ MVPA, and associated fitness and psychosocial factors at postintervention and at three months post intervention follow-up.

## Method

### Trial design

The HERizon Project was a twelve week, four-arm randomised controlled pilot trial evaluating the effect of a remote PA intervention on adolescent girls’ MVPA. Ethical approval for The HERizon Project was obtained from Liverpool John Moore’s Research Ethics Committee (20/SPS/042) and the study is registered with clinicaltrials.gov (reference: NCT04766372). The trial methods have been described in detail elsewhere (30–31). The design, implementation, and reporting of this study were guided by the Consolidated Standards of Reporting Trial checklist (CONSORT; Table S1 in Supplementary materials) [32] and the template for intervention description and replication checklist (TIDieR; Table S2 in Supplementary materials) [33]. Baseline (T0) data were collected remotely in December 2020 to January 2021. Immediately following this, the twelve-week intervention began. Postintervention (T1) data collection occurred one week after the intervention period between March to April 2021, and 3-month follow up (T2) data collection occurred 3 months following the end of the intervention between July to August 2021.

### Participants

Adolescent girls aged 13–16 years old, living in the UK or Ireland, seeking support in increasing their PA, were eligible to take part in the study. Adolescents who had a condition that prevented them from engaging in PA, were pregnant, or who did not have access to a smartphone or computer were excluded from taking part in the study. Participants were recruited via social media advertisements. Written informed parental/guardian consent, and adolescent assent were collected prior to randomisation and data collection.

After baseline measurements, participants were randomised with equal distribution to a (i) PA programme group, (ii) behaviour change support group, (iii) combined PA programme and behaviour change support group, or (iv) comparison group.

### Interventions

#### PA programme group

Participants in the PA programme arm (PROGRAM) received a PA logbook where they were asked to self-record their PA sessions each week. Participants were also invited to join two weekly live online group workouts, led by the first author (a certified group instructor with experience leading adolescent exercise). Workouts lasted approximately 35 min and began with a dynamic warm up, followed by bodyweight circuits including push ups, squats, and cardiovascular exercises, and finished with a static stretching cool down. To cater for all abilities and further embed autonomy and perceived competence, modifications were provided for all exercises to make movements easier or more difficult (e.g., to make squats easier, participants were asked to sit down onto a chair. To make the movement more difficult, participants were invited to hold tins or a water bottle to add further resistance). Although participants were invited to turn their camera on, the majority did not. Therefore, the instructor provided regular generic encouragement throughout the session and gave regular exercise cues to support correct and safe exercise form. As the live workout schedule did not suit all participants, sessions were recorded and made available online to re-play at a later time. Participants in this group were also sent three standardised non-reply text messages each week with reminders (e.g., “Live workouts this week are at 6.30pm Wednesday and 10am Saturday”), encouragement (e.g., “You are halfway through already! This is when motivation can start to dip but keep focused on your goals, you are doing incredibly”), and support (e.g., “How have you been getting on so far? If you have any questions, please make sure to send us an email at [researcher email address]”). Participants also had access to a private group chat on Instagram, moderated by the first author. This intervention component aimed to further support participant’s need for relatedness as it gave them an opportunity to interact with other girls in their group.

#### Behaviour change support group

Participants randomised into the behaviour change support arm (BC) also received a PA logbook where they recorded their weekly PA sessions. Participants in this group were partnered with an Activity Mentor for the duration of the intervention. Mentors (*n* = 12) were trainee sport and exercise psychology students (Master of psychology students and Professional Doctorate students), trained and supervised by a Health and Care Professions Council-registered Sport and Exercise Psychologist (second author). Participants met with their Activity Mentor via video call on week 0 for a 30-minute introduction, throughout the intervention on weeks 1–6 and week 9 for 15-minute calls, and on week 12 for a final 30-minute call. On these calls, participants told their Activity Mentor what PA they completed the previous week and made a PA plan for the coming week. Behaviour change support calls were grounded in SDT and aimed to foster participants’ autonomy, relatedness, and competence through needs-supportive delivery. To standardise support, Activity Mentors followed a pre-planned session guide which covered various topics each week (e.g., week one focused on action planning, week two focused on identifying barriers – Table S3 in supplementary material). To further support standardisation, all Activity Mentors completed a training workshop prior to the start of the intervention, received an intervention manual that outlined policies and procedures to deal with arising issues in a consistent way, and met for weekly group reflection meetings with the HCPC Sport and Exercise Psychologist (second author) and the lead researcher (first author).

#### Combined group

Participants in the combined arm (COMBO) received all intervention components outlined in the PA programme and behaviour change support sections above.

#### Comparison group

Participants in the comparison group only received a PA logbook (Figure S1 in Supplementary material) at the beginning of the intervention and did not have any further contact or support from the research team outside of data collection points at baseline, postintervention and 3-month follow up.

### Outcome measures and procedures

Demographic data were collected at baseline (T0), whilst physical activity, physical fitness and psychosocial data were collected at all assessment points; baseline (T0), postintervention (T1) and 3-month follow up (T2). Following consent, parents/guardians completed an online questionnaire that collected participant demographic data. Data included age, country, ethnicity, home postcode and menstruation status. The last three digits of home postcodes were mapped against national indices of deprivation (34–35) and participants were categorised into terciles of deprivation accordingly (most affluent to most deprived). Due to the COVID-19 pandemic, all outcome measures were completed by participants remotely following instruction from the research team, with assistance from parents/guardians as required.

#### Physical activity

Device-measured physical activity was assessed using Actigraph accelerometers (GT9X and GT3X + models, Pensacola, Florida, USA). Participants were asked to wear the device on their non-dominant wrist for nine days at baseline (week 0), one-week postintervention (week 13), and 3 months after the intervention had ended (week 24), only removing it when bathing, swimming or for safety reasons. The accelerometers recorded data at a frequency of 100 Hz over 1s epochs [36]. Participants received the device by post, along with an information sheet and wear time diary which was used to record times the device was taken off, as well as the time they woke up and went to sleep each day. Participants were required to wear the device for at least 10 h per day during waking hours [37], which were estimated to be between 6am and 11pm based on previous literature [38]. To be included in the final analysis, participants were required to have valid wear time on at least three weekdays and one weekend day [39]. Non-wear time was classified as periods lasting ≥ 60 min of consecutive zero values and this data was discarded [39]. To account for seasonal variations in participants’ PA, mean precipitation (cm), temperature (°C), and day length (hrs) were recorded for valid PA data at each time point [40] using the Met Office and Met Éireann websites. Using the ActiLife software (version 6.13-0, ActiGraph, Pensacola, FL, USA), raw accelerations were downloaded from the devices and exported into .cvs files. This raw data was then used to calculate time spent in MVPA using the GGIR work package [39, 41] from R Studio software (version 2.6-0, www.r-project.org*).* Using Hildebrand [42] cut-points, MVPA was classified as time accumulated in acceleration ≥ 201*mg*, as used previously with adolescent girls [10, 43]. To increase wear compliance, text message reminders were sent to participants each morning during data collection time points. Within each group and at each time point, participants who met wear-time criteria were entered into a draw for a £50 gift card.

Self-reported PA was assessed using the 8-item subscale of the World Health Organisation Health Behaviours of School aged Children (HBSC) questionnaire [44], which has been validated previously with adolescents [44, 45]. Following HBSC implementation instructions, the questionnaire was introduced with a short summary of what MVPA is (“*Physical activity is any activity that increases your heart rate and makes you get out of breath some of the time. Physical activity can be done in sports*,* school activities*,* playing with friends*,* or walking to school. Some examples include running*,* walking quickly*,* cycling”).* The first question assessed participants’ MVPA by asking “*Over the past 7 days*,* on how many days were you physically active for a total of at least 60 minutes per day?*” to which responses included 0 days to 7 days. To assess the frequency of participants’ leisure time vigorous PA, participants were asked “*How often do you usually exercise in your free time so much that you get out of breath or sweat?*” responses were “daily”, “4–6 times a week”, “2–3 times a week”, “once a week”, “once a month”, “less than a month”, and “never”. Answers were coded using a seven-point Likert scale. To assess the duration of participants’ leisure time vigorous PA, participants were asked *“How many hours a week do you usually exercise in your free time so much that you get out of breath or sweat?”* responses were “none”, “about half an hour”, “about an hour”, “about 2–3 hours”, “about 4–6 hours”, and ‘7 hours or more”. Answers were coded using a six-point Likert scale.

#### Physical fitness

‘Resistance Training for Teens’ (RTT) was used to collect participants’ fitness test scores (46–47). This is a free, password-protected mobile application that allowed the research team access to participants’ fitness data. Information on how to set up, perform, and record each test was outlined within the mobile application. Parents/guardians assisted participants in the set-up and conduction of the field-based fitness tests.

Cardiorespiratory fitness was measured by a 20-metre shuttle run test [48], which has been validated with adolescents [49]. This test required participants to run back and forth between two markers distanced 20 m apart, at a pace set by the audio file on the RTT application. Participants began running at a speed of 8.5 kmph and incrementally increased by 0.5 kmph as the levels progressed (approximately every minute). Parents/guardians were asked to provide verbal encouragement with the aim of participants reaching volatile exhaustion. The test was terminated when participants could no longer reach the marker for two consecutive beeps. The last successfully completed level was recorded in the mobile application.

Muscular endurance was measured by a push up test [50]. Push ups were performed from a plank position, with toes touching the floor. The chest was then lowered towards the ground until elbows were at a 90-degree angle from which participants then pushed back up into a plank position. Participants were asked to perform as many push ups as possible at a cadence of 40 bpm (as set by an audio file on the RTT application). Parents counted the number of push up repetitions, and when correct exercise form could no longer be maintained, the total number was recorded in the RTT application.

Muscular strength was measured by standing long jump test, which has been validated in adolescents [50]. Standing with toes behind a line or marker, participants performed a standing long jump, jumping from and landing on two feet. A parent/guardian then marked the landing and measured the distance between the two markers. Participants performed the jump twice, and the longest jump was recorded in the RTT application.

#### Psychosocial outcomes

Psychosocial questionnaires were completed online via Google Forms. To obtain an overall score for each questionnaire, individual questions were scored using the corresponding Likert scale and a mean value was then calculated.

The Behavioural Regulation in Exercise 3 (BREQ-3) was used to measure exercise motivation (a combination of BREQ-2 [51], four additional integrated regulation items [52], and one additional introjected regulation item [53]. Participants were scored using a 5-point Likert scale (“Not true” = 0, “True” = 4), which measured their amotivation, controlled motivation (external regulation and introjected regulation), and autonomous motivation (identified regulation, integrated regulation, and intrinsic motivation).

The Body Appreciation Scale was used as a measure of participants’ body image [54]. The 10-item questionnaire included statements such as “I feel love for my body” and was scored using a 5-point Likert scale (“Never” = 1, “Always” = 5), with high scores reflecting high body appreciation.

The Perceived Competence Scale [55] was used to measure participants’ perceived PA competence. The 4-item questionnaire included statements such as “I am capable of being physically active regularly” and was scored using a 7-point Likert scale (“Strongly disagree” = 1, “Strongly agree” = 7), with higher scores reflecting higher perceived competence in PA.

The Adolescent Self-Esteem Questionnaire was used to measure participants’ self-esteem [56]. The 12-item questionnaire included statements such as “I feel I can be myself around other people” and was scored using a 5-point Likert scale (“Hardly ever” = 1, “Almost all of the time” = 5), with higher scores reflecting higher self-esteem.

### Sample size and randomisation

Following the Medical Research Council guidelines on the development and evaluation of complex interventions [26], this study is classified as a feasibility trial. Using the median sample size of feasibility trials within the UK Clinical Research Network database [57], this study aimed to recruit 40 participants into each group (total of *N* = 160 participants). Once this sample size was reached, recruitment ended. Participants were randomly allocated with equal distribution into one of four groups, with country-level (UK and Ireland) stratification, using a computer-generated algorithm by the first author. Double-blind testing was not possible due to the nature of the study. To minimise contamination, cluster-randomisation was used for participants who enrolled with a sister/friend/classmate, therefore not all groups were allocated equal numbers of participants.

### Statistical methods

A linear mixed model was used to adjust for multiple observations between participants, and to assess the differences within and between groups. Models were used to assess change in each outcome variable between (a) baseline and postintervention, and (b) baseline and 3-month follow up. Analyses were modelled as a function of intervention group membership, controlling for age, country of residence, and deprivation status. Each outcome was modelled separately, and a complete case analysis was used (a case was defined by a combination of person and of time) (Table S4 in Supplementary materials). All statistical analyses were completed using SPSS for Mac (version 27, IBM, Armonk, NY, USA) and statistical significance was set at ≤ 0.05. Descriptive statistics are presented as the mean ± SD, unless stated otherwise, and outcomes of linear mixed models as the mean (95% CI).

## Results

A total of 153 participants were randomised into one of the four intervention arms. For full details on recruitment rates please see Cowley et al. [30] and Figure S2 in Supplementary Material. The mean age of participants was 14.8 years (standard deviation; SD, 1.4 years), 81% were of white ethnicity, 50% resided in the UK, and 34% lived in areas of low social deprivation (Table 1). Across the sample, participants’ compliance to wearing the accelerometers declined between data collection timepoints (75% compliance at baseline, 52% at postintervention, and 37% at 3-month follow up). Participant MVPA, physical fitness and psychosocial outcomes are presented in Table 2.

**Table 1:** Summary of participant baseline characteristics

Characteristics	PROGRAM (*n* = 35)	BC (*n* = 45)	COMBO (*n* = 37)	Comparison (*n* = 36)
Age, mean (SD), years	14.9 ± 1.6	14.9 ± 1.1	14.9 ± 1.2	14.8 ± 1.2
Reside in UK^1^, n (%)	15 (43%)	24 (53%)	23 (62%)	15 (42%)
Ethnicity, n (%)				
*White*	29 (83%)	36 (80%)	30 (81%)	29 (81%)
*Asian or Asian British/Irish*	4 (8%)	3 (7%)	5 (13%)	4 (11%)
*African/ Caribbean Black British/Irish*	2 (6%)	4 (9%)	1 (3%)	1 (3%)
*Mixed ethnic groups*	1 (3%)	2 (4%)	1 (3%)	2 (5%)
Socioeconomic status, *n* (%) ^a^				
*Tercile 1*	9 (26%)	10 (22%)	22 (59%)	11 (30%)
*Tercile 2*	24 (69%)	25 (57%)	11 (30%)	19 (53%)
*Tercile 3*	2 (5%)	10 (22%)	4 (11%)	6 (17%)
Accelerometer MVPA, min	33.2 ± 34.6	43.1 ± 67.1	39.42 ± 74.9	45.03 ± 55.8
HBSC MVPA^1^, days	2.2 ± 1.7	2.2 ± 1.7	2.4 ± 1.5	2.3 ± 1.8
HBSC VPA^2^, days	3.0 ± 1.4	3.1 ± 1.3	3.2 ± 1.4	3.3 ± 1.5
HBSC VPA^3^, hours	2.0 ± 1.4	2.2 ± 1.2	2.0 ± 1.2	1.9 ± 1.5
Push up, rep	5.3 ± 5.3	8.3 ± 7.8	7.9 ± 7.3	7.2 ± 7.4
Long jump, cm	156.6 ± 28.4	162.8 ± 28.4	155.3 ± 32.1	157.3 ± 30.1
Shuttle run, levels	5.2 ± 3.4	4.3 ± 2.3	4.8 ± 3.0	5.2 ± 3.3
Perceived competence	5.2 ± 1.1	5.1 ± 1.0	5.1 ± 1.2	5.1 ± 1.2
Body appreciation	3.4 ± 0.9	3.5 ± 0.9	3.1 ± 0.8	3.0 ± 0.9
Self esteem	3.5 ± 0.7	3.7 ± 0.6	3.4 ± 0.7	3.4 ± 0.7
Amotivation	0.7 ± 1.3	0.4 ± 0.6	0.7 ± 1.1	0.5 ± 1.0
External regulation	1.7 ± 1.7	1.4 ± 1.2	1.7 ± 1.5	1.5 ± 1.4
Introjected regulation	2.4 ± 1.7	2.3 ± 1.4	2.6 ± 1.4	2.9 ± 1.2
Identified regulation	3.6 ± 0.9	3.8 ± 0.7	3.6 ± 0.9	3.9 ± 0.9
Integrated regulation	2.2 ± 1.4	2.8 ± 1.1	2.5 ± 1.5	2.7 ± 1.4
Intrinsic motivation	3.6 ± 0.9	3.8 ± 1.1	3.4 ± 1.5	3.6 ± 1.3

Groups contained different sample sizes due to cluster randomisation. *Abbreviations*: BC behaviour change arm, PROGRAM physical activity arm, COMBO combined arm, ^1^UK United Kingdom, PA physical activity, MVPA moderate to vigorous physical activity, VPA vigorous physical activity, min minutes, rep repetition, cm centimetre, HBSC health behaviours of school children questionnaire. ^a^Socioeconomic status was determined based on home postcodes using the Irish Pobal HP Deprivation Index and the UK Index of Multiple Deprivations (1 = most deprived, 2 = median deprived, 3 = least deprived).^1^ frequency of accruing ≥ 60 min MVPA ^2^ frequency of VPA days per week ^3^duration of VPA hours per week.

### Baseline to postintervention

No significant differences were found between groups at postintervention for device-measured MVPA, self-reported HBSC questionnaires, or physical fitness tests. For psychosocial variables, significant differences were identified between groups for perceived competence (*P* = 0.028), integrated regulation (*P* = 0.016), and intrinsic motivation (*P* = 0.045).

Posthoc analyses (Table 3) found that perceived competence improved significantly more in the PROGRAM arm (*P* = 0.032, estimated mean difference of 0.6 points), BC arm (*P* = 0.024, estimated mean difference of 0.6 points), and COMBO arm (*P* = 0.004, estimated mean difference of 0.8 points) compared to the comparison group. Likewise, identified regulation improved significantly more in the PROGRAM arm compared with the BC arm (*P* = 0.028, estimated mean difference of 0.5 points), COMBO arm (*P* = 0.019, estimated mean difference of 0.5 points), and the comparison group (*P* = 0.005, estimated mean difference of 0.7 points). Finally, intrinsic motivation improved significantly more in the COMBO arm compared to the comparison group (*P* = 0.009, estimated mean difference of 0.9 points).

### Baseline to 3-month follow up

No significant differences were found between groups at 3-month follow up for device measured or questionnaire measured PA outcomes, nor any psychosocial outcome. The only outcome variable significantly different between groups at 3-month follow up was push up score (*P* = 0.04). Posthoc analysis found there was a significantly greater improvement in push up scores in the COMBO arm compared to the PROGRAM arm (*P* = 0.025, estimated mean difference of 4 repetitions), and in the COMBO arm compared the comparison group (*P* = 0.019, estimated mean difference of 5 repetitions).

**Table 2:** Outcomes for physical activity, physical fitness and psychosocial measures in intervention arms and comparison groups. Between baseline, postintervention, and 3-month follow up

Variable	PROGRAM	BC		COMBO	Comparison		Group * Time *P*	PROGRAM	BC	COMBO	Comparison	Group * Time *P*
	Adjusted mean difference (95% confidence intervals) between baseline and postintervention							Adjusted mean difference (95% confidence intervals) between baseline and 3-month follow up				
MVPA, min	-20.4 (-53.3, 12.4)	-7.1 (-33.1, 18.8)	10.3 (-19.3, 39.9)		-16.0 (-55.8, 23.8)		0.524	20.7 (-27.0, 68.4)	12.5 (-21.7, 46.7)	4.5 (-27.9, 36.9)	8.5 (-39.1, 56.1)	0.948
HBSC MVPA, days^1^	2.6 (1.7, 3.3)	2.0 (1.3, 2.6)	2.1 (1.3, 2.8)		1.8 (0.9, 2.6)		0.548	2.8 (1.9, 3.7)	2.4 (1.5, 3.1)	1.6 (0.8, 2.3)	2.7 (1.7, 3.6)	0.158
HBSC VPA, days^2^	1.1 (0.5, 1.6)	1.0 (0.5, 1.4)	1.2 (0.7, 1.6)		1.2 (0.5, 1.6)		0.957	1.1 (0.4, 1.8)	1.6 (0.9, 2.1)	1.0 (0.4, 1.6)	1.1 (0.3, 1.7)	0.551
HBSC VPA, hours^3^	1.1 (0.5, 1.6)	0.8 (0.4, 1.2)	1.1 (0.6, 1.6)		0.5 (-0.1, 1.0)		0.294	1.5 (0.8, 2.0)	0.9 (0.3, 1.3)	0.9 (0.3, 1.4)	0.6 (0.0, 1.2)	0.177
Push up, rep	4 (2, 6)	4 (3, 6)	6 (4, 8)		4 (1, 6)		0.239	4 (1, 7)	7 (5, 9)	8 (5, 11)	4 (0, 6)	0.040*
Long jump, cm	16.5 (6.1, 26.9)	11.9 (3.7, 20.1)	8.0 (-1.4, 17.4)		9.9 (-0.6, 20.6)		0.630	17.2 (4.0, 30.3)	13.3 (2.6, 23.9)	4.1 (-7.9, 16.1)	16.6 (3.7, 29.4)	0.412
Shuttle run, levels	1.3 (0.6, 1.9)	1.2 (0.6, 1.6)	0.9 (0.2, 1.4)		1.0 (0.3, 1.7)		0.675	2.2 (1.4, 3.0)	1.8 (1.1, 2.4)	1.3 (0.5, 2.0)	0.9 (-0.1, 1.7)	0.073
Perceived competence	0.9 (0.5, 1.3)	0.9 (0.6, 1.3)	1.1 (0.7, 1.5)		0.3 (-0.1, 0.7)		0.028*	0.7 (0.1, 1.3)	0.8 (0.2, 1.3)	0.9 (0.3, 1.4)	0.4 (-0.2, 1.0)	0.704
Body appreciation	0.7 (0., 0.9)	0.3 (0.1, 0.5)	0.4 (0.1, 0.6)		0.2 (-0.1, 0.4)		0.072	0.5 (0.1, 0.8)	0.3 (-0.1, 0.5)	0.5 (0.2, 0.8)	0.3 (-0.1, 0.6)	0.400
Self esteem	0.4 (0.2, 0.6)	0.1 (-0.0, 0.3)	0.3 (0.1, 0.4)		0.2 (0.0, 0.4)		0.153	0.3 (0.1, 0.4)	0.1 (-0.1, 0.2)	0.3 (0.1, 0.5)	0.3 (0.1, 0.5)	0.266
Amotivation	-0.4 (-0.8, -0.0)	0.1 (-0.3, 0.2)	-0.2 (-0.5, 0.1)		-0.1 (-0.4, 0.3)		0.399	-0.4 (-0.8, 0.1)	-0.2 (-0.5, 0.2)	-0.5 (-0.8, -0.1)	-0.3 (-0.8, 0.1)	0.684
External regulation	-0.8 (-1.3, -0.1)	0.1 (-0.3, 0.5)	-0.4 (-0.7, 0.2)		-0.2 (-0.9, 0.2)		0.167	-0.4 (-1.0, 0.1)	-0.1 (-0.6, 0.4)	-0.5 (-0.9, 0.1)	-0.5 (-1.1, 0.1)	0.683
Introjected regulation	0 (-0.5, 0.4)	0.2 (-0.1, 0.5)	0 (-0.4, 0.4)		0.3 (-0.1, 0.8)		0.683	0.1 (-0.4, 0.7)	-0.1 (-0.5, 0.4)	-0.2 (-0.7, 0.2)	-0.3 (-0.8, 0.3)	0.669
Identified regulation	0.9 (0.5, 1.1)	0.4 (0.1, 0.6)	0.4 (0.0, 0.6)		0.2 (-0.1, 0.5)		0.016*	0.9 (0.4, 1.2)	0.5 (0.1, 0.8)	0.2 (-0.1, 0.5)	0.5 (0.1, 0.9)	0.074
Integrated regulation	1.2 (0.7, 1.7)	0.6 (0.1, 0.9)	0.7 (0.2, 1.1)		0.4 (-0.0, 0.9)		0.094	0.7 (0.1, 1.3)	0.3 (-0.1, 0.8)	0.6 (0.1, 1.2)	0.6 (-0.1, 1.2)	0.757
Intrinsic motivation	1.1 (0.6, 1.6)	0.5 (0.1, 0.9)	0.9 (0.3, 1.3)		0.2 (-0.2, 0.7)		0.045*	0.8 (0.2, 1.2)	0.3 (-0.1, 0.7)	0.8 (0.4, 1.2)	0.4 (-0.1, 0.8)	0.194

Between-group differences significant at * *P* < 0.05. *Abbreviations* BC: behaviour change arm; PROGRAM: physical activity arm; COMBO: combined arm; PA: physical activity; MVPA: moderate to vigorous physical activity; VPA: vigorous physical activity; min: minutes; rep: repetition; cm: centimetre; HBSC: health behaviours of school children questionnaire. ).^1^ frequency of accruing ≥ 60 min MVPA ^2^ frequency of VPA days per week ^3^duration of VPA hours per week

**Table 3:** Post-hoc analyses and change scores for significantly different outcome variables between intervention arms

	Between baseline (T0) and postintervention (T1)						Between postintervention (T1) and 3-month follow up (T2)	
	Competence		Identified regulation		Intrinsic motivation		Push Ups	
	*Estimated mean difference (ES)*	Group * time P	*Estimated mean difference (ES)*	Group * time P	*Estimated mean difference (ES)*	Group * time P	*Estimated mean difference (ES)*	Group * time P
BC vs. Comparison	0.6 (0.63)	0.025*	0.2 (0.28)	0.309	0.3 (0.25)	0.370	4 (0.65)	0.054
PROGRAM vs. Comparison	0.6 (0.61)	0.032*	0.7 (0.88)	0.002*	0.9 (0.75)	0.009*	0 (0.07)	0.828
COMBO vs. Comparison	0.8 (0.83)	0.004*	0.2 (0.21)	0.444	0.6 (0.52)	0.069	5 (0.82)	0.0019*
BC vs. PROG	0 (0.01)	0.955	-0.5 (-0.59)	0.028*	-0.6 (-0.50)	0.063	3 (0.58)	0.067
BC vs. COMBO	-0.2 (-0.12)	0.433	0 (0.06)	0.796	-0.3 (-0.27)	0.288	-1 (-0.16)	0.588
PROGRAM vs. COMB	-0.2 (-0.21)	0.445	0.5 (0.66)	0.019*	0.3 (0.23)	0.408	-4 (-0.74)	0.025*

Between-group differences significant at * *P* < 0.05. *Abbreviations* ES effect size Cohen’s d, BC behaviour change arm, PROGRAM physical activity arm, COMBO combined arm

## Discussion

This study aimed to examine the preliminary effectiveness of specific HERizon intervention components on increasing adolescent girls’ MVPA, and associated fitness and psychosocial factors. It further aimed to assess if changes in outcome measures were sustained three months following the end of the intervention period. Results demonstrate that there were significant improvements in perceived competence in all intervention arms compared to the comparison group at postintervention, with the greatest increase in the PA programme group. There were also significant improvements in identified regulation and intrinsic motivation in the PA programme group compared to the other intervention arms and comparison group. At 3-month follow up, the only significant increase was found in muscular endurance in the behaviour change and combined groups, with the combined group showing the greatest improvement in push up score. No changes were found in device-measured and self-reported PA at either timepoint.

In line with our previous formative evaluation of a smaller scale HERizon trial [29], no differences were found for PA outcome measures. Increasing adolescent girls’ PA levels is a difficult task, evidenced by the plethora of previous interventions resulting in no significant changes between baseline and postintervention [9, 12, 23, 58]. To promote participant autonomy, no one form of PA was enforced during HERizon e.g., some participants took up yoga, others joined circuit-based group workouts, and some decided to join sports teams. It is hypothesised that this variance in PA type, duration, and intensity, alongside wrist-worn accelerometers inability to detect certain activities [59], negated the overall group level intervention effect on MVPA. Further, certain types of PA are more likely to improve certain fitness outcomes than others [60] e.g., participants who took up jogging were likely to improve shuttle run scores but unlikely to see improvements in their push up score. When outcome and process evaluation results are taken together, a more holistic view on the effectiveness of an intervention can be drawn [26]. From the contextual information gained through the HERizon process evaluation [30], we know that the intervention was ending at the same time COVID-19 lockdown restrictions were being lifted. Further, the intervention started during the school term but ended as participants were starting their summer holidays. Given the majority of adolescent PA is gained during the structured school day [61], it is possible this change in daily routine is another important contributor to poor accelerometer compliance rates and large variation in the MVPA data.

Echoing results of the previous formative evaluation [30], this larger scale trial also found significant improvements in autonomous behaviours at postintervention. Unexpectedly, girls randomised to the PA programme group had greatest improvements in identified regulation and intrinsic motivation. Although qualitative feedback demonstrates the value participants place on working with an Activity Mentor [30], results of the current study suggest that one-to-one behaviour change coaching was not more beneficial than a group-based intervention in improving autonomous forms of motivation. Further, a combined approach was also not found more beneficial as participants in both groups attended the same number of live group workouts and similar engagement rates in the online community chat. As this was a pilot trial with relatively small group sample size, it is difficult to draw definitive explanations to this finding. It is interesting to note that participants in the PA programme group were more likely to complete all PA sessions and use their PA logbook than participants in any other group [30], perhaps contributing to greater autonomous behaviours at postintervention. Although the reasons behind these group differences are unclear, it is encouraging that participation in certain intervention arms were associated with psychological benefits which may facilitate future engagement in PA. A recent study by Rodrigues and colleagues [62] found that people who perceived themselves as having positive exercise experiences, in which their basic psychological needs were met, had a higher probability of engaging in future exercise. Therefore, it may be important for interventions to first prioritise improving girls’ perception of PA through increasing autonomy, competence, and relatedness as a prerequisite to increasing their MVPA levels. Intrinsically motivated people engage in PA for the pure joy, satisfaction, and absorption in the activity, rather than due to guilt or external rewards [19]. Further, intrinsic motivation is one of the strongest predictors of long-term PA adherence [63]. Therefore, it is plausible that a longer intervention and follow up may have resulted in increased PA levels and/or physical fitness scores due to a sustained enjoyment, and thus engagement in PA [62].

HERizon messaging focused on self-improvement, rather than on comparison with others. In line with our formative work [28], a recent systematic review found that girls often disengage from PA when they compare their skills to peers, and that low motivation towards PA often stems from girls’ low perceived competence [20]. During postintervention focus groups, girls spoke about enjoying PA more as they became fitter and more confident in their physical skills over the course of the intervention [30]. At the end of the intervention, girls in all intervention arms had increased perceived competence scores in contrast to the comparison group. These results are in line with previous research that found that as girls’ perceived competence and confidence increased, they were more likely to be motivated to improve skills and reach personal goals (20, 64–65). In contrast to much past literature discussing girls’ dislike of sweating and being untidy during exercise (66–67), participants spoke of enjoying the challenge of difficult workouts and feeling proud of their newfound strength and stamina [30]. Exercising in the privacy of home in a structured PA programme gave girls an opportunity to increase their perceived competence by developing skills without being fearful of others judging them. It is suggested that by allowing girls to exercise at home, they can experience exercises, particularly exercise traditionally considered “not for girls” (e.g., weightlifting and boxing), sheltered from the gender boundaries typically evident in PE settings [68–69].

### Limitations

As with many interventions implemented in uncontrolled “real-world” settings, there were several limitations to the research, specifically regarding accelerometer non-adherence, poor retention towards the end of the intervention period, and a short follow up period. Although participants were sent daily text message reminders to wear their wrist-worn accelerometer, as well as being given incentives to comply with the wear instructions, less than half of participants had valid accelerometer data at postintervention and a third of participants had valid data at 3-month follow up. This was a major limitation of the study as device-measured MVPA was the primary outcome. Secondly, as the twelve-week programme progressed, participants began to disengage, i.e., compared to week one of the intervention, fewer participants attended the live workout sessions, and more participants who were partnered with an Activity Mentor did not attend their video call sessions. Results from exit surveys suggest that many of the participants were sitting national school examinations towards the end of the intervention period, while others were going abroad on summer vacation [30]. It is possible that if the intervention was longer, the change in participants routines would not have resulted in such a disengagement. Further, it is possible that the support provided to participants by the Activity Mentors was weaned away too quickly (i.e., after only 6 weekly sessions). A longer intervention period would have allowed for a slower transition to independence and a more established PA routine. Lastly, due to pragmatic issues, it was not possible to have a longer follow up period than three months. As outlined above, a longer follow up period may have identified more sustained positive results. Further, the follow up data collection point occurred during summer break from school. Implementing this data collection during the school term would have been more appropriate as this would have been comparable to baseline data collection.

### Implications and future directions

Learnings from the HERizon intervention should be considered when designing future studies and interventions targeting adolescent girls’ PA levels. Results suggest that one-to-one behaviour change support does not add anything more than a group PA programme in improving intrinsic motivation towards PA. This has important implications as a more scalable and cost-effective intervention could be implemented by removing this individualised aspect. A larger trial is now needed to confirm these results and to assess the effectiveness of HERizon on girls’ long-term engagement in PA and their motivation towards being active. Specifically, further investigation is needed to explore if there is an association between increased intrinsic motivation and perceived competence, and long-term increases in MVPA and physical fitness.

HERizon also has implications for schools, clubs, and other initiatives aiming to increase girls’ PA. Although we cannot draw firm conclusions on the effectiveness of specific intervention components, it is clear that providing girls choice in the types of PA and exercise is critical to participant enjoyment [30]. In particular, girls should be introduced to unconventional types of exercise, typically not on physical education curricula, to demonstrate there are more ways to be active than through traditional team-based sports. Finally, creating a supportive and non-judgemental environment in which girls feel supported is essential in getting participant “buy-in”. Using positive language, having relatable instructors, and using individual goal setting were successful components of HERizon that contributed to fostering a supportive culture.

## Conclusion

This study was part of a larger body of work investigating the impact of a multi-component, remote intervention aimed at improving adolescent girls’ PA. This study focused on exploring which intervention components had the greatest impact on increasing girls’ MVPA. Results suggest that HERizon had no significant effect on increasing girls’ MVPA at postintervention or 3-month follow up, although it is important to consider the small sample size. Girls in all intervention arms had increased intrinsic motivation and competence scores at postintervention, and participants in the combined intervention arm had increased muscular endurance at 3-month follow up. Future research is needed to better understand these between-group differences and to explore if there is a correlation between improved autonomous motivation and long-term PA behaviour.

### Electronic supplementary material

Below is the link to the electronic supplementary material.

Supplementary Material 1

## Data Availability

This trial was part of a larger body of work, The HERizon Project, and has been registered with clinicaltrials.gov (registration number NCT04766372 and registration date 16/12/2020). The dataset generated and analysed during this current study is available in the Zenodo public repository (https://zenodo.org/doi/10.5281/zenodo.10379513).
