# Supplementary material for: Remote physical activity intervention to promote physical activity and health in adolescent girls (the HERizon project): a multi-arm, pilot randomised trial
Source: BMC Public Health. 2024 Aug 3;24:2098. doi: 10.1186/s12889-024-19664-7 (PMC11297741; doi:10.1186/s12889-024-19664-7)
Supplement: Supplementary file 1 — Supplementary Material 1 [file 12889_2024_19664_MOESM1_ESM.docx]

**Table S1:** The HERizon Project details in accordance with CONSORT checklist for pilot trials

| **Section** | **Item** | **Checklist item** | **Page** |
| --- | --- | --- | --- |
| **Title and abstract** | | | |
|  | 1a | Identification as a pilot or feasibility randomised trial in the title | 1 |
|  | 1b | Structured summary of pilot trial design, methods, results, and conclusions (for specific guidance see CONSORT abstract extension for pilot trials) | 2 |
| **Introduction** | | | |
| Background & objectives | 2a | Scientific background and explanation of rationale for future definitive trial, and reasons for randomised pilot trial | 4 |
|  | 2b | Specific objectives or research questions for pilot trial | 5 |
| **Methods** | | | |
| Trial design | 3a | Description of pilot trial design (such as parallel, factorial) including allocation ratio | 5 |
|  | 3b | Important changes to methods after pilot trial commencement (such as eligibility criteria), with reasons | N/A |
| Participants | 4a | Eligibility criteria for participants | 5 |
|  | 4b | Settings and locations where the data were collected | 5 |
|  | 4c | How participants were identified and consented | 5-6 |
| Interventions | 5 | The interventions for each group with sufficient details to allow replication, including how and when they were actually administered | 6-7 |
| Outcomes | 6a | Completely defined prespecified assessments or measurements to address each pilot trial objective specified in 2b, including how and when they were assessed | 8-11 |
|  | 6b | Any changes to pilot trial assessments or measurements after the pilot trial commenced, with reasons | N/A |
|  | 6c | If applicable, prespecified criteria used to judge whether, or how, to proceed with future definitive trial | N/A |
| Sample size | 7a | Rationale for numbers in the pilot trial | 11 |
|  | 7b | When applicable, explanation of any interim analyses and stopping guidelines | N/A |
| *Randomisation* | | | |
| Sequence generation | 8a | Method used to generate the random allocation sequence | 11 |
|  | 8b | Type of randomisation(s); details of any restriction (such as blocking and block size) | 11 |
| Allocation concealment mechanism | 9 | Mechanism used to implement the random allocation sequence (such as sequentially numbered containers), describing any steps taken to conceal the sequence until interventions were assigned | 11 |
| Implementation | 10 | Who generated the random allocation sequence, who enrolled participants, and who assigned participants to interventions | 11 |
| Blinding | 11a | If done, who was blinded after assignment to interventions (for example, participants, care providers, those assessing outcomes) and how | 11 |
|  | 11b | If relevant, description of the similarity of interventions | N/A |
| Statistical methods | 12 | Methods used to address each pilot trial objective whether qualitative or quantitative | 11 |
| **Results** | | | |
| Participant flow | 13a | For each group, the numbers of participants who were approached and/or assessed for eligibility, randomly assigned, received intended treatment, and were assessed for each objective | 12 |
|  | 13b | For each group, losses and exclusions after randomisation, together with reasons | 12 |
| Recruitment | 14a | Dates defining the periods of recruitment and follow-up | 5 |
|  | 14b | Why the pilot trial ended or was stopped | 11 |
| Baseline data | 15 | A table showing baseline demographic and clinical characteristics for each group | 12-13 |
| Numbers analysed | 16 | For each objective, number of participants (denominator) included in each analysis. If relevant, these numbers should be by randomised group | 12-13 |
| Outcomes & estimation | 17 | For each objective, results including expressions of uncertainty (such as 95% confidence interval) for any estimates. If relevant, these results should be by randomised group | 16 |
| Ancillary analyses | 18 | Results of any other analyses performed that could be used to inform the future definitive trial | 17 |
| Harms | 19 | All important harms or unintended effects in each group (for specific guidance see CONSORT for harms) | NA |
|  | 19a | If relevant, other important unintended consequences | 19 |
| **Discussion** | | | |
| Limitations | 20 | Pilot trial limitations, addressing sources of potential bias and remaining uncertainty about feasibility | 20-21 |
| Generalisability | 21 | Generalisability (applicability) of pilot trial methods and findings to future definitive trial and other studies | 21-22 |
| Interpretation | 22 | Interpretation consistent with pilot trial objectives and findings, balancing potential benefits and harms, and  considering other relevant evidence | 18-19 |
|  | 22a | Implications for progression from pilot to future definitive trial, including any proposed amendments | 21-22 |
| **Other information** | | | |
| Registration | 23 | Registration number for pilot trial and name of trial registry | 5 |
| Protocol | 24 | Where the pilot trial protocol can be accessed, if available | N/A |
| Funding | 25 | Sources of funding and other support (such as supply of drugs), role of funders | 27 |
| Ethics | 26 | Ethical approval or approval by research review committee, confirmed with reference number | 5 |

**Table S2**: The HERizon Project details in accordance with the TIDieR checklist.

| **#** | **Item** | **Page** |
| --- | --- | --- |
| 1 | **Brief name** Provide the name or phrase that describes the intervention | 5 |
| 2 | **Why** Describe the rational, theory, or goal or the elements essential to the intervention | 5 |
| 3 | **What** *Materials*: describe any physical or informational materials used in the intervention, including those provided to participants or used in intervention delivery or in training of intervention providers. Provide information on where the materials can be accessed (online appendix, URL). | 6-7 |
| 4 | *Procedures*: describe each of the procedures, activities, and/or processes used in the intervention, including any enabling or support activities. | 6-7 |
| 5 | **Who provided** For each category of intervention provider (e.g. psychologist, nursing assistance), describe their expertise, background and any specific training given. | 6-7 |
| 6 | **How** Describe the models of delivery (e.g. face-to-face or by some other mechanism, such as internet or telephone) of the intervention and whether it was provided individually or in a group. | 6-7 |
| 7 | **Where** Describe the type(s) of location(s) where the intervention occurred, including any necessary infrastructure or relevant features. | 5-10 |
| 8 | **When and how much** Describe the number of times the intervention was delivered and over what time period including the number of sessions, their schedule and their duration, intensity or dose | 5-7 |
| 9 | **Tailoring** If the intervention was planned to be personalised, titrated, or adapted, then describe what, why, when and how. | N/A |
| 10 | **Modifications**: If the intervention was modified during the study, describe the changes (what, why, when and how). | N/A |
| 11 | **How well** *Planned*: if intervention adherence or fidelity was assessed, describe how and by whom, and if any strategies were used to maintain or improve fidelity, describe them | N/A |
| 12 | **How well** *Actual*: if intervention adherence or fidelity was assessed, describe the extent to which the intervention was delivered as planned | 12 |

**Table S3.** Overview of 12-week delivery framework for Activity Mentor behaviour change support calls.

| **Week** | **Description of session** | |
| --- | --- | --- |
| 0 (30 min) | Introduction – rapport building and goal setting | |
| 1 | Setting action plans | |
| 2 | Barrier identification | |
| 3 | Action plan review (no specific topic) | Review & adjust action plan |
| 4 | Action plan review (no specific topic) |  |
| 5 | Coping planning - Part A |  |
| 6 | Reflect on achievements |  |
| 9 | Coping planning – Part B |  |
| 12 (30 min) | Final reflection |  |

**Table S4.** Outcomes for physical activity, physical fitness and psychosocial measures in intervention arms and comparison groups. Between baseline and postintervention including complete case analysis data.

|  | *Group* | *Estimated mean difference (time 1 vs. 0)* | *Pr > F* | *n* | *95% CI* | *Effect size* |
| --- | --- | --- | --- | --- | --- | --- |
| Average MVPA | Comparison | -16.0291 | 0.5243 | 12 | -55.887, 23.829 |  |
|  | Behaviour change | -7.1244 |  | 23 | -33.107, 18.858 |  |
|  | PA programme | -20.4356 |  | 15 | -53.351, 12.479 |  |
|  | Combined | 10.2848 |  | 17 | -19.358, 39.928 |  |
|  | Comparison vs. Behaviour change | -8.9047 | 0.6967 |  | -54.393, 36.584 | -0.15180 |
|  | Comparison vs. PA programme | 4.4065 | 0.8518 |  | -42.571, 51.384 | 0.07512 |
|  | Comparison vs. Combined | -26.3138 | 0.2757 |  | -74.174, 21.546 | -0.44857 |
|  | Behaviour change vs. PA programme | 13.3112 | 0.5133 |  | -27.189, 53.812 | 0.22692 |
|  | Behaviour change vs. Combined | -17.4092 | 0.3741 |  | -56.303, 21.485 | -0.29678 |
|  | PA programme vs. Combined | -30.7204 | 0.1601 |  | -73.933, 12.493 | -0.52369 |
| Push ups | Comparison | 3.4687 | 0.2399 | 19 | 1.166, 5.772 |  |
|  | Behaviour change | 4.2299 |  | 38 | 2.685, 5.775 |  |
|  | PA programme | 3.8756 |  | 27 | 1.891, 5.861 |  |
|  | Combined | 5.9989 |  | 29 | 4.200, 7.797 |  |
|  | Comparison vs. Behaviour change | -0.7611 | 0.5812 |  | -3.489, 1.966 | -0.16351 |
|  | Comparison vs. PA programme | -0.4068 | 0.7724 |  | -3.189, 2.375 | -0.08740 |
|  | Comparison vs. Combined | -2.5301 | 0.0749 |  | -5.318, 0.258 | -0.54355 |
|  | Behaviour change vs. PA programme | 0.3543 | 0.7735 |  | -2.081, 2.789 | 0.07611 |
|  | Behaviour change vs. Combined | -1.7690 | 0.1378 |  | -4.114, 0.576 | -0.38004 |
|  | PA programme vs. Combined | -2.1233 | 0.1044 |  | -4.693, 0.447 | -0.45615 |
| Longjump | Comparison | 9.9623 | 0.6305 | 24 | -0.676, 20.601 |  |
|  | Behaviour change | 11.9594 |  | 35 | 3.790, 20.129 |  |
|  | PA programme | 16.5092 |  | 27 | 6.106, 26.912 |  |
|  | Combined | 8.0314 |  | 27 | -1.435, 17.498 |  |
|  | Comparison vs. Behaviour change | -1.9971 | 0.7642 |  | -15.165, 11.170 | -0.08444 |
|  | Comparison vs. PA programme | -6.5469 | 0.3328 |  | -19.888, 6.794 | -0.27680 |
|  | Comparison vs. Combined | 1.9309 | 0.7805 |  | -11.774, 15.636 | 0.08164 |
|  | Behaviour change vs. PA programme | -4.5497 | 0.4802 |  | -17.282, 8.182 | -0.19236 |
|  | Behaviour change vs. Combined | 3.9281 | 0.5303 |  | -8.442, 16.298 | 0.16608 |
|  | PA programme vs. Combined | 8.4778 | 0.2179 |  | -5.084, 22.039 | 0.35844 |
| Shuttle run | Comparison | 1.0223 | 0.6751 | 20 | 0.316, 1.728 |  |
|  | Behaviour change | 1.1972 |  | 35 | 0.697, 1.698 |  |
|  | PA programme | 1.3312 |  | 26 | 0.699, 1.964 |  |
|  | Combined | 0.8508 |  | 26 | 0.253, 1.449 |  |
|  | Comparison vs. Behaviour change | -0.1749 | 0.6863 |  | -1.032, 0.682 | -0.11957 |
|  | Comparison vs. PA programme | -0.3089 | 0.4831 |  | -1.180, 0.562 | -0.21119 |
|  | Comparison vs. Combined | 0.1715 | 0.7040 |  | -0.722, 1.065 | 0.11722 |
|  | Behaviour change vs. PA programme | -0.1340 | 0.7370 |  | -0.924, 0.656 | -0.09161 |
|  | Behaviour change vs. Combined | 0.3464 | 0.3744 |  | -0.424, 1.117 | 0.23679 |
|  | PA programme vs. Combined | 0.4804 | 0.2563 |  | -0.355, 1.315 | 0.32841 |
| Self-reported MVPA | Comparison | 1.8245 | 0.5489 | 23 | 0.977, 2.672 |  |
|  | Behaviour change | 2.0239 |  | 36 | 1.382, 2.666 |  |
|  | PA programme | 2.5811 |  | 27 | 1.770, 3.393 |  |
|  | Combined | 2.0813 |  | 29 | 1.354, 2.808 |  |
|  | Comparison vs. Behaviour change | -0.1995 | 0.7094 |  | -1.258, 0.859 | -0.10415 |
|  | Comparison vs. PA programme | -0.7566 | 0.1707 |  | -1.844, 0.331 | -0.39502 |
|  | Comparison vs. Combined | -0.2568 | 0.6407 |  | -1.344, 0.831 | -0.13407 |
|  | Behaviour change vs. PA programme | -0.5572 | 0.2801 |  | -1.574, 0.460 | -0.29087 |
|  | Behaviour change vs. Combined | -0.0573 | 0.9066 |  | -1.023, 0.908 | -0.02992 |
|  | PA programme vs. Combined | 0.4998 | 0.3553 |  | -0.567, 1.567 | 0.26095 |
| Self-reported VPA frequency | Comparison | 1.0607 | 0.9574 | 24 | 0.514, 1.608 |  |
|  | Behaviour change | 1.0086 |  | 35 | 0.581, 1.436 |  |
|  | PA programme | 1.1180 |  | 27 | 0.586, 1.650 |  |
|  | Combined | 1.1808 |  | 29 | 0.701, 1.661 |  |
|  | Comparison vs. Behaviour change | 0.0521 | 0.8815 |  | -0.639, 0.744 | 0.04141 |
|  | Comparison vs. PA programme | -0.0573 | 0.8724 |  | -0.763, 0.648 | -0.04550 |
|  | Comparison vs. Combined | -0.1201 | 0.7375 |  | -0.829, 0.589 | -0.09543 |
|  | Behaviour change vs. PA programme | -0.1094 | 0.7480 |  | -0.783, 0.564 | -0.08691 |
|  | Behaviour change vs. Combined | -0.1723 | 0.5956 |  | -0.814, 0.469 | -0.13684 |
|  | PA programme vs. Combined | -0.0629 | 0.8597 |  | -0.766, 0.641 | -0.04993 |
| Self-reported VPA duration | Comparison | 0.4800 | 0.2944 | 24 | -0.081, 1.041 |  |
|  | Behaviour change | 0.8598 |  | 36 | 0.427, 1.293 |  |
|  | PA programme | 1.0581 |  | 27 | 0.512, 1.604 |  |
|  | Combined | 1.1187 |  | 29 | 0.627, 1.610 |  |
|  | Comparison vs. Behaviour change | -0.3797 | 0.2881 |  | -1.085, 0.325 | -0.29423 |
|  | Comparison vs. PA programme | -0.5780 | 0.1160 |  | -1.301, 0.145 | -0.44789 |
|  | Comparison vs. Combined | -0.6386 | 0.0843 |  | -1.365, 0.088 | -0.49484 |
|  | Behaviour change vs. PA programme | -0.1983 | 0.5674 |  | -0.884, 0.487 | -0.15365 |
|  | Behaviour change vs. Combined | -0.2589 | 0.4341 |  | -0.913, 0.395 | -0.20061 |
|  | PA programme vs. Combined | -0.0606 | 0.8680 |  | -0.782, 0.660 | -0.04695 |
| Competence | Comparison | 0.3268 | 0.0284 | 24 | -0.115, 0.769 |  |
|  | Behaviour change | 0.9647 |  | 36 | 0.624, 1.305 |  |
|  | PA programme | 0.9493 |  | 27 | 0.520, 1.379 |  |
|  | Combined | 1.1666 |  | 30 | 0.786, 1.547 |  |
|  | Comparison vs. Behaviour change | -0.6380 | 0.0246 |  | -1.193, -0.083 | -0.62819 |
|  | Comparison vs. PA programme | -0.6226 | 0.0323 |  | -1.192, -0.054 | -0.61305 |
|  | Comparison vs. Combined | -0.8399 | 0.0041 |  | -1.407, -0.273 | -0.82699 |
|  | Behaviour change vs. PA programme | 0.0154 | 0.9550 |  | -0.524, 0.555 | 0.01515 |
|  | Behaviour change vs. Combined | -0.2019 | 0.4329 |  | -0.710, 0.306 | -0.19880 |
|  | PA programme vs. Combined | -0.2173 | 0.4451 |  | -0.779, 0.345 | -0.21395 |
| Body appreciation | Comparison | 0.1705 | 0.0721 | 24 | -0.118, 0.459 |  |
|  | Behaviour change | 0.3056 |  | 36 | 0.083, 0.528 |  |
|  | PA programme | 0.6519 |  | 27 | 0.371, 0.933 |  |
|  | Combined | 0.3748 |  | 30 | 0.126, 0.623 |  |
|  | Comparison vs. Behaviour change | -0.1351 | 0.4616 |  | -0.497, 0.227 | -0.20361 |
|  | Comparison vs. PA programme | -0.4814 | 0.0116 |  | -0.853, -0.110 | -0.72564 |
|  | Comparison vs. Combined | -0.2043 | 0.2770 |  | -0.575, 0.166 | -0.30793 |
|  | Behaviour change vs. PA programme | -0.3463 | 0.0539 |  | -0.699, 0.006 | -0.52203 |
|  | Behaviour change vs. Combined | -0.0692 | 0.6804 |  | -0.401, 0.263 | -0.10432 |
|  | PA programme vs. Combined | 0.2771 | 0.1375 |  | -0.090, 0.644 | 0.41771 |
| Self esteem | Comparison | 0.2243 | 0.1538 | 24 | 0.005, 0.444 |  |
|  | Behaviour change | 0.1361 |  | 36 | -0.033, 0.305 |  |
|  | PA programme | 0.4342 |  | 27 | 0.221, 0.648 |  |
|  | Combined | 0.3024 |  | 30 | 0.114, 0.491 |  |
|  | Comparison vs. Behaviour change | 0.0881 | 0.5272 |  | -0.187, 0.364 | 0.17481 |
|  | Comparison vs. PA programme | -0.2100 | 0.1436 |  | -0.492, 0.073 | -0.41643 |
|  | Comparison vs. Combined | -0.0781 | 0.5836 |  | -0.360, 0.204 | -0.15495 |
|  | Behaviour change vs. PA programme | -0.2981 | 0.0294 |  | -0.566, -0.030 | -0.59124 |
|  | Behaviour change vs. Combined | -0.1663 | 0.1944 |  | -0.419, 0.086 | -0.32976 |
|  | PA programme vs. Combined | 0.1318 | 0.3510 |  | -0.147, 0.411 | 0.26148 |
| Amotivation | Comparison | -0.0599 | 0.3996 | 24 | -0.475, 0.355 |  |
|  | Behaviour change | -0.0583 |  | 36 | -0.378, 0.262 |  |
|  | PA programme | -0.4425 |  | 27 | -0.846, -0.039 |  |
|  | Combined | -0.2359 |  | 30 | -0.593, 0.121 |  |
|  | Comparison vs. Behaviour change | -0.0016 | 0.9952 |  | -0.523, 0.520 | -0.00167 |
|  | Comparison vs. PA programme | 0.3826 | 0.1589 |  | -0.152, 0.917 | 0.40096 |
|  | Comparison vs. Combined | 0.1760 | 0.5142 |  | -0.357, 0.709 | 0.18443 |
|  | Behaviour change vs. PA programme | 0.3842 | 0.1357 |  | -0.122, 0.891 | 0.40263 |
|  | Behaviour change vs. Combined | 0.1776 | 0.4628 |  | -0.300, 0.655 | 0.18610 |
|  | PA programme vs. Combined | -0.2066 | 0.4396 |  | -0.735, 0.321 | -0.21653 |
| External motivation | Comparison | -0.3727 | 0.1678 | 24 | -0.967, 0.222 |  |
|  | Behaviour change | 0.0738 |  | 36 | -0.384, 0.532 |  |
|  | PA programme | -0.7511 |  | 27 | -1.329, -0.173 |  |
|  | Combined | -0.2173 |  | 30 | -0.729, 0.294 |  |
|  | Comparison vs. Behaviour change | -0.4465 | 0.2380 |  | -1.192, 0.299 | -0.32698 |
|  | Comparison vs. PA programme | 0.3784 | 0.3291 |  | -0.387, 1.143 | 0.27712 |
|  | Comparison vs. Combined | -0.1554 | 0.6871 |  | -0.918, 0.607 | -0.11381 |
|  | Behaviour change vs. PA programme | 0.8249 | 0.0261 |  | 0.100, 1.550 | 0.60410 |
|  | Behaviour change vs. Combined | 0.2911 | 0.4005 |  | -0.392, 0.975 | 0.21317 |
|  | PA programme vs. Combined | -0.5338 | 0.1642 |  | -1.289, 0.222 | -0.39093 |
| Introject motivation | Comparison | 0.3040 | 0.6838 | 24 | -0.197, 0.805 |  |
|  | Behaviour change | 0.1965 |  | 36 | -0.190, 0.583 |  |
|  | PA programme | -0.0413 |  | 27 | -0.529, 0.446 |  |
|  | Combined | 0.0252 |  | 30 | -0.406, 0.456 |  |
|  | Comparison vs. Behaviour change | 0.1076 | 0.7354 |  | -0.522, 0.737 | 0.09338 |
|  | Comparison vs. PA programme | 0.3453 | 0.2912 |  | -0.300, 0.991 | 0.29983 |
|  | Comparison vs. Combined | 0.2788 | 0.3923 |  | -0.365, 0.922 | 0.24208 |
|  | Behaviour change vs. PA programme | 0.2378 | 0.4425 |  | -0.374, 0.849 | 0.20645 |
|  | Behaviour change vs. Combined | 0.1713 | 0.5572 |  | -0.405, 0.748 | 0.14870 |
|  | PA programme vs. Combined | -0.0665 | 0.8365 |  | -0.704, 0.571 | -0.05774 |
| Identified regulation | Comparison | 0.1979 | 0.0167 | 24 | -0.133, 0.529 |  |
|  | Behaviour change | 0.4116 |  | 36 | 0.157, 0.666 |  |
|  | PA programme | 0.8637 |  | 27 | 0.542, 1.185 |  |
|  | Combined | 0.3621 |  | 30 | 0.078, 0.647 |  |
|  | Comparison vs. Behaviour change | -0.2138 | 0.3095 |  | -0.629, 0.201 | -0.28135 |
|  | Comparison vs. PA programme | -0.6658 | 0.0025 |  | -1.091, -0.240 | -0.87630 |
|  | Comparison vs. Combined | -0.1642 | 0.4449 |  | -0.589, 0.260 | -0.21608 |
|  | Behaviour change vs. PA programme | -0.4520 | 0.0284 |  | -0.855, -0.049 | -0.59496 |
|  | Behaviour change vs. Combined | 0.0496 | 0.7966 |  | -0.331, 0.430 | 0.06527 |
|  | PA programme vs. Combined | 0.5016 | 0.0198 |  | 0.081, 0.922 | 0.66023 |
| Integrated regulation | Comparison | 0.4411 | 0.0944 | 24 | -0.076, 0.958 |  |
|  | Behaviour change | 0.5665 |  | 36 | 0.168, 0.965 |  |
|  | PA programme | 1.2242 |  | 27 | 0.722, 1.727 |  |
|  | Combined | 0.6747 |  | 30 | 0.230, 1.119 |  |
|  | Comparison vs. Behaviour change | -0.1254 | 0.7024 |  | -0.774, 0.523 | -0.10558 |
|  | Comparison vs. PA programme | -0.7831 | 0.0215 |  | -1.448, -0.118 | -0.65941 |
|  | Comparison vs. Combined | -0.2335 | 0.4868 |  | -0.897, 0.430 | -0.19666 |
|  | Behaviour change vs. PA programme | -0.6577 | 0.0410 |  | -1.288, -0.027 | -0.55383 |
|  | Behaviour change vs. Combined | -0.1082 | 0.7191 |  | -0.703, 0.486 | -0.09108 |
|  | PA programme vs. Combined | 0.5495 | 0.1003 |  | -0.108, 1.207 | 0.46275 |
| Intrinsic motivation | Comparison | 0.2271 | 0.0457 | 24 | -0.297, 0.751 |  |
|  | Behaviour change | 0.5254 |  | 36 | 0.122, 0.929 |  |
|  | PA programme | 1.1292 |  | 27 | 0.620, 1.639 |  |
|  | Combined | 0.8500 |  | 30 | 0.399, 1.301 |  |
|  | Comparison vs. Behaviour change | -0.2983 | 0.3706 |  | -0.956, 0.359 | -0.24774 |
|  | Comparison vs. PA programme | -0.9021 | 0.0092 |  | -1.577, -0.227 | -0.74919 |
|  | Comparison vs. Combined | -0.6229 | 0.0692 |  | -1.295, 0.050 | -0.51731 |
|  | Behaviour change vs. PA programme | -0.6038 | 0.0639 |  | -1.243, 0.035 | -0.50145 |
|  | Behaviour change vs. Combined | -0.3246 | 0.2882 |  | -0.927, 0.278 | -0.26956 |
|  | PA programme vs. Combined | 0.2792 | 0.4080 |  | -0.387, 0.945 | 0.23188 |

**Table S5.** Outcomes for physical activity, physical fitness and psychosocial measures in intervention arms and comparison groups. Between baseline and 3-month follow up including complete case analysis data.

|  | *Group* | *Estimated mean difference (time 2 vs. 0)* | *Pr > F* | *n* | *95% CI* | *Effect size* |
| --- | --- | --- | --- | --- | --- | --- |
| Average MVPA | Comparison | 8.5089 | 0.9483 | 10 | -39.173, 56.191 |  |
|  | Behaviour change | 12.4963 |  | 15 | -21.779, 46.772 |  |
|  | PA programme | 20.7049 |  | 9 | -27.029, 68.439 |  |
|  | Combined | 4.4947 |  | 16 | -27.963, 36.952 |  |
|  | Comparison vs. Behaviour change | -3.9874 | 0.8890 |  | -61.300, 53.325 | -0.06253 |
|  | Comparison vs. PA programme | -12.1959 | 0.6989 |  | -75.384, 50.992 | -0.19125 |
|  | Comparison vs. Combined | 4.0142 | 0.8877 |  | -52.983, 61.011 | 0.06295 |
|  | Behaviour change vs. PA programme | -8.2086 | 0.7753 |  | -65.861, 49.444 | -0.12872 |
|  | Behaviour change vs. Combined | 8.0016 | 0.7311 |  | -38.674, 54.677 | 0.12548 |
|  | PA programme vs. Combined | 16.2101 | 0.5721 |  | -41.238, 73.658 | 0.25420 |
| Push ups | Comparison | 3.4774 | 0.0409 | 17 | 0.490, 6.465 |  |
|  | Behaviour change | 7.0929 |  | 26 | 4.841, 9.345 |  |
|  | PA programme | 3.8822 |  | 19 | 1.112, 6.653 |  |
|  | Combined | 7.9945 |  | 20 | 5.477, 10.512 |  |
|  | Comparison vs. Behaviour change | -3.6155 | 0.0540 |  | -7.295, 0.064 | -0.65456 |
|  | Comparison vs. PA programme | -0.4047 | 0.8286 |  | -4.116, 3.306 | -0.07327 |
|  | Comparison vs. Combined | -4.5170 | 0.0193 |  | -8.281, -0.753 | -0.81779 |
|  | Behaviour change vs. PA programme | 3.2107 | 0.0678 |  | -0.241, 6.662 | 0.58129 |
|  | Behaviour change vs. Combined | -0.9016 | 0.5888 |  | -4.210, 2.407 | -0.16323 |
|  | PA programme vs. Combined | -4.1123 | 0.0257 |  | -7.710, -0.514 | -0.74452 |
| Longjump | Comparison | 16.6353 | 0.4120 | 20 | 3.790, 29.481 |  |
|  | Behaviour change | 13.2927 |  | 25 | 2.650, 23.935 |  |
|  | PA programme | 17.1913 |  | 19 | 4.017, 30.365 |  |
|  | Combined | 4.1091 |  | 18 | -7.934, 16.152 |  |
|  | Comparison vs. Behaviour change | 3.3425 | 0.6866 |  | -13.099, 19.784 | 0.13207 |
|  | Comparison vs. PA programme | -0.5560 | 0.9466 |  | -17.027, 15.915 | -0.02197 |
|  | Comparison vs. Combined | 12.5261 | 0.1497 |  | -4.618, 29.670 | 0.49495 |
|  | Behaviour change vs. PA programme | -3.8985 | 0.6324 |  | -20.071, 12.274 | -0.15404 |
|  | Behaviour change vs. Combined | 9.1836 | 0.2498 |  | -6.592, 24.959 | 0.36287 |
|  | PA programme vs. Combined | 13.0821 | 0.1346 |  | -4.148, 30.312 | 0.51692 |
| Shuttle run | Comparison | 0.8582 | 0.0738 | 15 | -0.013, 1.729 |  |
|  | Behaviour change | 1.8077 |  | 25 | 1.164, 2.452 |  |
|  | PA programme | 2.2370 |  | 18 | 1.441, 3.033 |  |
|  | Combined | 1.3303 |  | 19 | 0.592, 2.069 |  |
|  | Comparison vs. Behaviour change | -0.9495 | 0.0810 |  | -2.019, 0.120 | -0.60979 |
|  | Comparison vs. PA programme | -1.3788 | 0.0146 |  | -2.477, -0.281 | -0.88548 |
|  | Comparison vs. Combined | -0.4721 | 0.3976 |  | -1.578, 0.634 | -0.30319 |
|  | Behaviour change vs. PA programme | -0.4293 | 0.3910 |  | -1.421, 0.563 | -0.27568 |
|  | Behaviour change vs. Combined | 0.4774 | 0.3238 |  | -0.481, 1.436 | 0.30661 |
|  | PA programme vs. Combined | 0.9067 | 0.0856 |  | -0.131, 1.944 | 0.58229 |
| Self-reported MVPA | Comparison | 2.6921 | 0.1582 | 19 | 1.745, 3.639 |  |
|  | Behaviour change | 2.3558 |  | 25 | 1.558, 3.153 |  |
|  | PA programme | 2.8220 |  | 20 | 1.900, 3.744 |  |
|  | Combined | 1.5883 |  | 24 | 0.807, 2.370 |  |
|  | Comparison vs. Behaviour change | 0.3363 | 0.5833 |  | -0.879, 1.551 | 0.17826 |
|  | Comparison vs. PA programme | -0.1299 | 0.8326 |  | -1.348, 1.089 | -0.06884 |
|  | Comparison vs. Combined | 1.1038 | 0.0690 |  | -0.088, 2.296 | 0.58508 |
|  | Behaviour change vs. PA programme | -0.4662 | 0.4282 |  | -1.631, 0.699 | -0.24710 |
|  | Behaviour change vs. Combined | 0.7675 | 0.1678 |  | -0.330, 1.865 | 0.40681 |
|  | PA programme vs. Combined | 1.2337 | 0.0404 |  | 0.056, 2.412 | 0.65392 |
| Self-reported VPA frequency | Comparison | 1.0935 | 0.5516 | 19 | 0.394, 1.793 |  |
|  | Behaviour change | 1.5736 |  | 24 | 0.974, 2.174 |  |
|  | PA programme | 1.1330 |  | 20 | 0.450, 1.816 |  |
|  | Combined | 1.0102 |  | 23 | 0.419, 1.601 |  |
|  | Comparison vs. Behaviour change | -0.4801 | 0.2952 |  | -1.387, 0.427 | -0.34374 |
|  | Comparison vs. PA programme | -0.0395 | 0.9308 |  | -0.942, 0.863 | -0.02829 |
|  | Comparison vs. Combined | 0.0833 | 0.8528 |  | -0.807, 0.974 | 0.05961 |
|  | Behaviour change vs. PA programme | 0.4406 | 0.3172 |  | -0.431, 1.312 | 0.31545 |
|  | Behaviour change vs. Combined | 0.5634 | 0.1819 |  | -0.269, 1.396 | 0.40335 |
|  | PA programme vs. Combined | 0.1228 | 0.7828 |  | -0.761, 1.007 | 0.08790 |
| Self-reported VPA duration | Comparison | 0.6198 | 0.1771 | 19 | 0.007, 1.232 |  |
|  | Behaviour change | 0.8537 |  | 25 | 0.336, 1.372 |  |
|  | PA programme | 1.4686 |  | 20 | 0.871, 2.066 |  |
|  | Combined | 0.9121 |  | 23 | 0.395, 1.429 |  |
|  | Comparison vs. Behaviour change | -0.2339 | 0.5563 |  | -1.022, 0.554 | -0.19131 |
|  | Comparison vs. PA programme | -0.8488 | 0.0355 |  | -1.639, -0.059 | -0.69428 |
|  | Comparison vs. Combined | -0.2923 | 0.4576 |  | -1.072, 0.487 | -0.23909 |
|  | Behaviour change vs. PA programme | -0.6149 | 0.1090 |  | -1.370, 0.140 | -0.50297 |
|  | Behaviour change vs. Combined | -0.0584 | 0.8727 |  | -0.781, 0.665 | -0.04778 |
|  | PA programme vs. Combined | 0.5565 | 0.1560 |  | -0.217, 1.330 | 0.45519 |
| Competence | Comparison | 0.4007 | 0.7047 | 19 | -0.258, 1.059 |  |
|  | Behaviour change | 0.8206 |  | 25 | 0.266, 1.375 |  |
|  | PA programme | 0.7429 |  | 20 | 0.102, 1.384 |  |
|  | Combined | 0.8581 |  | 24 | 0.315, 1.401 |  |
|  | Comparison vs. Behaviour change | -0.4198 | 0.3256 |  | -1.264, 0.425 | -0.32011 |
|  | Comparison vs. PA programme | -0.3422 | 0.4238 |  | -1.189, 0.505 | -0.26089 |
|  | Comparison vs. Combined | -0.4573 | 0.2753 |  | -1.286, 0.371 | -0.34869 |
|  | Behaviour change vs. PA programme | 0.0777 | 0.8491 |  | -0.732, 0.888 | 0.05922 |
|  | Behaviour change vs. Combined | -0.0375 | 0.9224 |  | -0.800, 0.725 | -0.02858 |
|  | PA programme vs. Combined | -0.1152 | 0.7803 |  | -0.934, 0.704 | -0.08780 |
| Body appreciation | Comparison | 0.2582 | 0.4008 | 19 | -0.089, 0.605 |  |
|  | Behaviour change | 0.2886 |  | 25 | -0.004, 0.581 |  |
|  | PA programme | 0.5246 |  | 20 | 0.187, 0.863 |  |
|  | Combined | 0.5331 |  | 24 | 0.247, 0.820 |  |
|  | Comparison vs. Behaviour change | -0.0305 | 0.8921 |  | -0.476, 0.415 | -0.04405 |
|  | Comparison vs. PA programme | -0.2664 | 0.2386 |  | -0.713, 0.180 | -0.38531 |
|  | Comparison vs. Combined | -0.2750 | 0.2140 |  | -0.712, 0.162 | -0.39767 |
|  | Behaviour change vs. PA programme | -0.2360 | 0.2747 |  | -0.663, 0.191 | -0.34126 |
|  | Behaviour change vs. Combined | -0.2445 | 0.2299 |  | -0.647, 0.158 | -0.35362 |
|  | PA programme vs. Combined | -0.0085 | 0.9687 |  | -0.440, 0.423 | -0.01235 |
| Self esteem | Comparison | 0.3151 | 0.2668 | 19 | 0.087, 0.543 |  |
|  | Behaviour change | 0.0856 |  | 25 | -0.107, 0.278 |  |
|  | PA programme | 0.2528 |  | 20 | 0.030, 0.475 |  |
|  | Combined | 0.3308 |  | 24 | 0.142, 0.519 |  |
|  | Comparison vs. Behaviour change | 0.2295 | 0.1230 |  | -0.063, 0.522 | 0.50446 |
|  | Comparison vs. PA programme | 0.0623 | 0.6741 |  | -0.231, 0.356 | 0.13696 |
|  | Comparison vs. Combined | -0.0156 | 0.9140 |  | -0.303, 0.272 | -0.03440 |
|  | Behaviour change vs. PA programme | -0.1672 | 0.2397 |  | -0.448, 0.114 | -0.36750 |
|  | Behaviour change vs. Combined | -0.2451 | 0.0689 |  | -0.510, 0.019 | -0.53886 |
|  | PA programme vs. Combined | -0.0780 | 0.5865 |  | -0.362, 0.206 | -0.17136 |
| Amotivation | Comparison | -0.3409 | 0.6848 | 19 | -0.800, 0.118 |  |
|  | Behaviour change | -0.1709 |  | 25 | -0.557, 0.215 |  |
|  | PA programme | -0.3947 |  | 20 | -0.842, 0.052 |  |
|  | Combined | -0.4884 |  | 24 | -0.867, -0.110 |  |
|  | Comparison vs. Behaviour change | -0.1700 | 0.5672 |  | -0.759, 0.419 | -0.18596 |
|  | Comparison vs. PA programme | 0.0538 | 0.8566 |  | -0.537, 0.644 | 0.05884 |
|  | Comparison vs. Combined | 0.1475 | 0.6127 |  | -0.430, 0.725 | 0.16131 |
|  | Behaviour change vs. PA programme | 0.2238 | 0.4325 |  | -0.341, 0.788 | 0.24480 |
|  | Behaviour change vs. Combined | 0.3175 | 0.2383 |  | -0.214, 0.849 | 0.34727 |
|  | PA programme vs. Combined | 0.0937 | 0.7449 |  | -0.477, 0.665 | 0.10247 |
| External motivation | Comparison | -0.5440 | 0.6834 | 19 | -1.174, 0.086 |  |
|  | Behaviour change | -0.1017 |  | 25 | -0.632, 0.429 |  |
|  | PA programme | -0.4353 |  | 20 | -1.049, 0.178 |  |
|  | Combined | -0.4532 |  | 24 | -0.973, 0.066 |  |
|  | Comparison vs. Behaviour change | -0.4423 | 0.2793 |  | -1.250, 0.366 | -0.35250 |
|  | Comparison vs. PA programme | -0.1087 | 0.7903 |  | -0.919, 0.702 | -0.08659 |
|  | Comparison vs. Combined | -0.0907 | 0.8204 |  | -0.883, 0.702 | -0.07231 |
|  | Behaviour change vs. PA programme | 0.3337 | 0.3940 |  | -0.441, 1.108 | 0.26591 |
|  | Behaviour change vs. Combined | 0.3516 | 0.3406 |  | -0.378, 1.081 | 0.28019 |
|  | PA programme vs. Combined | 0.0179 | 0.9638 |  | -0.766, 0.802 | 0.01428 |
| Introject motivation | Comparison | -0.2813 | 0.6692 | 19 | -0.867, 0.304 |  |
|  | Behaviour change | -0.0758 |  | 25 | -0.569, 0.417 |  |
|  | PA programme | 0.1360 |  | 20 | -0.434, 0.706 |  |
|  | Combined | -0.2442 |  | 24 | -0.727, 0.239 |  |
|  | Comparison vs. Behaviour change | -0.2054 | 0.5877 |  | -0.957, 0.546 | -0.17616 |
|  | Comparison vs. PA programme | -0.4172 | 0.2736 |  | -1.170, 0.336 | -0.35776 |
|  | Comparison vs. Combined | -0.0370 | 0.9206 |  | -0.774, 0.700 | -0.03175 |
|  | Behaviour change vs. PA programme | -0.2118 | 0.5600 |  | -0.932, 0.508 | -0.18160 |
|  | Behaviour change vs. Combined | 0.1684 | 0.6226 |  | -0.510, 0.847 | 0.14441 |
|  | PA programme vs. Combined | 0.3802 | 0.3020 |  | -0.348, 1.108 | 0.32601 |
| Identified motivation | Comparison | 0.5131 | 0.0747 | 19 | 0.093, 0.933 |  |
|  | Behaviour change | 0.4658 |  | 25 | 0.112, 0.820 |  |
|  | PA programme | 0.8877 |  | 20 | 0.479, 1.297 |  |
|  | Combined | 0.1854 |  | 24 | -0.161, 0.532 |  |
|  | Comparison vs. Behaviour change | 0.0473 | 0.8618 |  | -0.492, 0.586 | 0.05650 |
|  | Comparison vs. PA programme | -0.3746 | 0.1716 |  | -0.915, 0.166 | -0.44766 |
|  | Comparison vs. Combined | 0.3277 | 0.2210 |  | -0.201, 0.856 | 0.39156 |
|  | Behaviour change vs. PA programme | -0.4219 | 0.1081 |  | -0.939, 0.095 | -0.50416 |
|  | Behaviour change vs. Combined | 0.2804 | 0.2551 |  | -0.206, 0.767 | 0.33506 |
|  | PA programme vs. Combined | 0.7023 | 0.0091 |  | 0.180, 1.225 | 0.83922 |
| Integrated motivation | Comparison | 0.5845 | 0.7571 | 19 | -0.034, 1.203 |  |
|  | Behaviour change | 0.3327 |  | 25 | -0.188, 0.854 |  |
|  | PA programme | 0.7037 |  | 20 | 0.101, 1.306 |  |
|  | Combined | 0.6507 |  | 24 | 0.140, 1.161 |  |
|  | Comparison vs. Behaviour change | 0.2518 | 0.5297 |  | -0.542, 1.046 | 0.20427 |
|  | Comparison vs. PA programme | -0.1192 | 0.7666 |  | -0.915, 0.677 | -0.09666 |
|  | Comparison vs. Combined | -0.0662 | 0.8661 |  | -0.845, 0.713 | -0.05372 |
|  | Behaviour change vs. PA programme | -0.3710 | 0.3350 |  | -1.132, 0.390 | -0.30093 |
|  | Behaviour change vs. Combined | -0.3181 | 0.3801 |  | -1.035, 0.399 | -0.25799 |
|  | PA programme vs. Combined | 0.0529 | 0.8915 |  | -0.717, 0.823 | 0.04295 |
| Intrinsic motivation | Comparison | 0.3588 | 0.1941 | 19 | -0.154, 0.871 |  |
|  | Behaviour change | 0.3084 |  | 25 | -0.123, 0.740 |  |
|  | PA programme | 0.7568 |  | 20 | 0.258, 1.256 |  |
|  | Combined | 0.8447 |  | 24 | 0.422, 1.267 |  |
|  | Comparison vs. Behaviour change | 0.0504 | 0.8791 |  | -0.607, 0.708 | 0.04939 |
|  | Comparison vs. PA programme | -0.3980 | 0.2330 |  | -1.057, 0.261 | -0.39000 |
|  | Comparison vs. Combined | -0.4859 | 0.1376 |  | -1.131, 0.159 | -0.47605 |
|  | Behaviour change vs. PA programme | -0.4485 | 0.1606 |  | -1.079, 0.182 | -0.43939 |
|  | Behaviour change vs. Combined | -0.5363 | 0.0760 |  | -1.130, 0.057 | -0.52544 |
|  | PA programme vs. Combined | -0.0878 | 0.7846 |  | -0.725, 0.550 | -0.08605 |


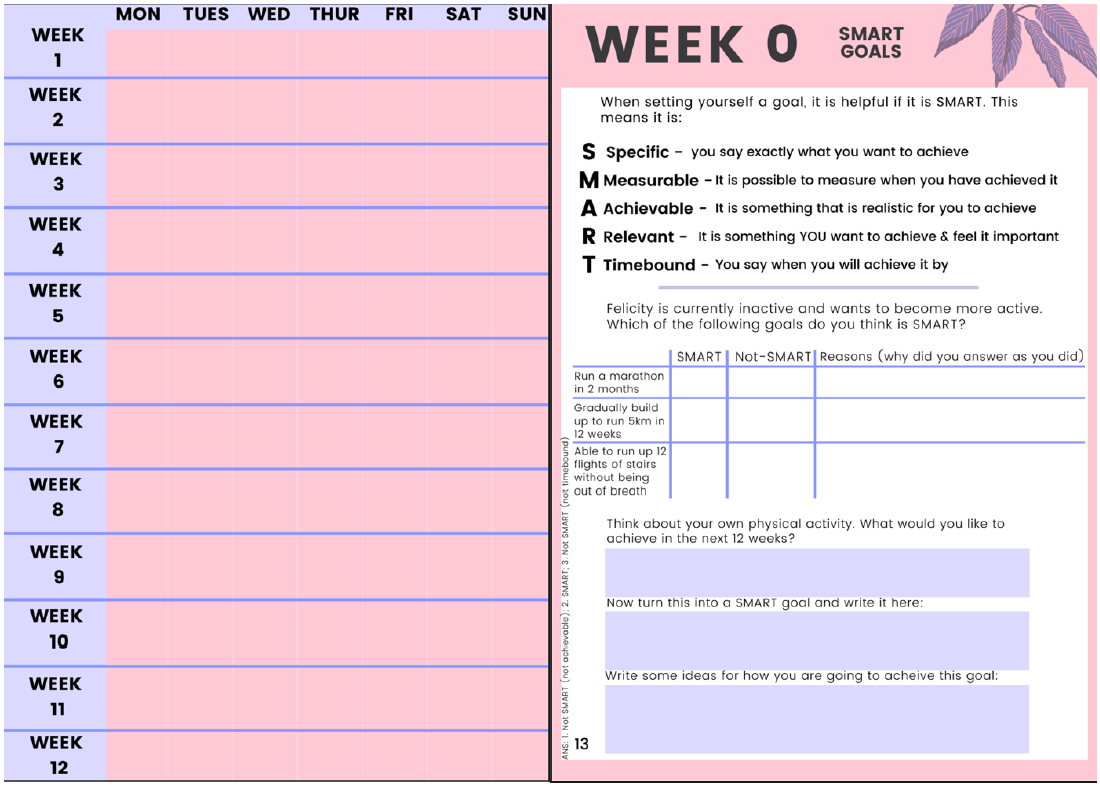


**Figure S1.** Sample pages from HERizon PA logbook

Nov – Dec 2020

**Randomisation**

**3 month follow up assessment T2**

**Baseline assessment T0**

*n* = 161

PA programme group

(*n* = 35)

Behaviour change support group (*n* = 45)

Combined group

(*n* = 37)

No reply, *n* = 3

COVID related, *n* = 2

School related, *n* = 1

Personal issue, *n* = 2

Comparison group

(*n* = 36)

**12-week HERizon intervention**

**Postintervention assessment** **T1**

Dec 2020 – Jan 2021

Jan - April 2021

July - Aug 2021

March - April 2021

PA programme group

(*n* = 31)

Behaviour change support group (*n* = 40)

Combined group

(*n* = 33)

Comparison group

(*n* = 27)

PA programme group

(*n* = 25)

Behaviour change support group (*n* = 32)

Combined group

(*n* = 27)

Comparison group

(*n* = 23)

**Figure S2.** CONSORT flow diagram of participant recruitment and withdrawal. Participants are considered withdrawn from a timepoint if they completed no outcome measures. If participants completed some outcome measures but not all, they are still included in the sample size of their group and timepoint.
